# Real-time imaging of bacterial colony growth dynamics for cells with Type IV-A1 CRISPR-Cas activity

**DOI:** 10.1093/femsml/uqaf006

**Published:** 2025-04-01

**Authors:** Selina Rust, Lennart Randau

**Affiliations:** Prokaryotic RNA Biology, Department of Biology, Philipps-Universität Marburg, D-35043 Marburg, Germany; Prokaryotic RNA Biology, Department of Biology, Philipps-Universität Marburg, D-35043 Marburg, Germany; SYNMIKRO, Center for Synthetic Microbiology, D-35043 Marburg, Germany

**Keywords:** microbial colonies, real-time imaging, colony heterogeneity, *E. coli*, CRISPR-Cas systems, CRISPRi

## Abstract

The Type IV-A1 CRISPR-Cas system of *Pseudomonas oleovorans* provides defense against mobile genetic elements in the absence of target DNA degradation. In recent studies, *Escherichia coli* BL21-AI cells with Type IV-A1 CRISPR-Cas activity displayed a heterogeneous colony growth phenotype. Here, we developed a convenient smartphone-mediated automatic remote-controlled time-lapse imaging system (SMARTIS), that enables monitoring of growing bacteria over time. The system’s design includes a custom-built imaging box equipped with LED lights, an adjustable heating system and a smartphone that can be remotely controlled using freely available, user-friendly applications. SMARTIS allowed long-term observation of growing colonies and was utilized to analyze different growth behaviors of *E. coli* cells expressing Type IV-A1 CRISPR ribonucleoproteins. Our findings reveal that heterogeneity in colonies can emerge within hours of initial growth. We further examined the influence of different expression systems on bacterial growth and CRISPR interference activity and demonstrated that the observed heterogeneity of colony-forming units is strongly influenced by plasmid design and backbone identity. This study highlights the importance of careful assessment of heterogenous colony growth dynamics and describes a real-time imaging system with wide applications beyond the study of CRISPR-Cas activity in bacterial hosts.

## Teaser

This study establishes a smartphone-mediated time-lapse imaging system (SMARTIS) to examine bacterial colony growth dynamics and utilizes this approach to analyze heterogenous growth of *Escherichia coli* cells expressing a Type IV-A1 CRISPR-Cas system.

## Introduction

The discovery of CRISPR-Cas systems highly impacted the field of genetic engineering, offering robust tools for precise genome editing. CRISPR (clustered regularly interspaced short palindromic repeats) systems consist of nonrepetitive spacer sequences, derived from invading DNA (protospacers), that are flanked by short palindromic repeats. Additionally, *cas* (CRISPR-associated) genes can be usually found in close vicinity to a CRISPR array (Barrangou et al. 2007, Horvath and Barrangou 2010, Makarova et al. 2011). CRISPR-Cas systems adapt to and fight against mobile genetic elements (MGEs), and are present in a wide range of bacteria and most archaea (Brouns et al. 2008, Sorek et al. 2008). Among the diverse CRISPR-Cas systems, the Type IV-A systems are characterized by a core set of Cas proteins that mediate plasmid clearance and gene regulation via a CRISPRi-like mechanism (Crowley et al. 2019, Özcan et al. 2019, Guo et al. 2022, Benz et al. 2024, Sanchez-Londono et al. 2024). Thus, unlike other CRISPR-Cas systems, Type IV-A does not cleave its target DNA, but is proposed to block transcription or transcription initiation. The presence of a protospacer adjacent motif (PAM) allows the host to identify foreign target DNA and enables defense against a wide variety of MGEs (Guo et al. 2022, Sanchez-Londono et al. 2024).

Our recent studies focused on the analysis of the Type IV-A1 CRISPR-Cas system found in *Pseudomonas oleovorans* strain DSM1045 (Guo et al. 2022, Sanchez-Londono et al. 2024). The system was shown to mediate interference against genomic, plasmid, and phage DNA. Here, endpoint images of LB agar plates were examined, in which colonies were evaluated for their number and color. Heterogeneity of colonies after *lacZ*-targeting assays was observed, but not further investigated (Guo et al. 2022). However, examination of the growth of macroscopically visible colonies is important to draw significant conclusions, providing a broader perspective on microbial population dynamics, community interactions, and the impact of environmental factors on bacterial growth (Warren et al. 2019).

Colony morphology, size, and distribution can indicate differences in metabolic activity, virulence, or resistance mechanisms across the population. Moreover, analyzing whole colonies can reveal patterns of competition among bacteria and allows to follow the influence of spatial organization on growth and survival.

While there are several systems able to visualize colonies at single time-points or over time (Clarke et al. 2010, Brugger et al. 2012, Peñil Cobo et al. 2018), the analysis of colony growth dynamics usually is characterized by high cost and technical complexity. The systems often require sophisticated equipment, such as high-resolution cameras, environmental control chambers for colony growth, and software interfaces that are often complex. Thus, affordable and user-friendly solutions for time-lapse imaging of bacterial colonies are needed. In recent years, efforts have been made to develop cost-effective systems for studying bacterial colonies. A microbial chamber (MOCHA) and various fluorescence microscopy methods were developed to allow imaging of bacterial colony growth, while preventing dehydration and condensation during long-term imaging (Peñil Cobo et al. 2018, Molina-Santiago et al. 2022). Additionally, an inexpensive imaging box was designed to document colonies at specific time-points (Smith and Schuster 2021). Further methods focused on the use of off-the-shelf components, such as smartphones, 3D-printed parts, and simple mechanical setups to achieve affordable high-quality imaging (Hernández Vera et al. 2016, Schaefer et al. 2023). By combining affordability with ease of use, these platforms have the potential to access advanced imaging technologies. However, these methods are often limited in their ability to grow bacteria at optimal temperatures. Thus, we created the smartphone-mediated automatic remote-controlled time-lapse imaging system (SMARTIS), a convenient approach designed to maintain optimal growth temperatures while being cost-effective and user friendly.

We used SMARTIS to capture the growth of *Escherichia coli* colonies after different CRISPR activity assays with the Type IV-A1 CRISPR-Cas system of *P. oleovorans* and were able to uncover potential causes for heterogenous growth of colony-forming units (CFU).

## Materials and methods

### Culture conditions and strains

*Escherichia coli* DH5α and *E. coli* BL21-AI were cultivated in LB media at 37°C, optionally supplemented with appropriate antibiotics.

### Design and generation of CRISPR-Cas plasmids

A derivative of a pETDuet™-1 plasmid (pSR13) was created to encode for the Type IV-A1 CRISPR-Cas associated genes from *P. oleovorans* DSM 1045 (GenBank: NIUB00000000.1). Another plasmid (pSR24) expresses the appropriate crRNA-precursor. Here, the CRISPR array carries a synthetic spacer sequence (32 bp) comprising recognition sites of the Type IIS restriction endonuclease BseRI, flanked by repeat sequences from the native Type IV-A1 CRISPR array. The CRISPR array and associated spacer sequences (targeting either pSR14 or the *lacZ* gene) were cloned according to Rust and Randau (2024). Generated plasmids were transformed into chemically competent *E. coli* DH5α cells and subsequently plated on Lysogeny Broth (LB) agar plates supplemented with 100 µg/ml ampicillin and 100 µg/ml spectinomycin. Successful cloning was confirmed by Polymerase Chain Reaction (PCR) using primers flanking the insertion site. The integration was further verified through Sanger sequencing. A detailed overview of plasmids used in this study can be found in Table S1.

### SMARTIS

Our SMARTIS is based on the use of a smartphone with suitable applications. The smartphone used in this study (Samsung Galaxy S7 mini) contains a camera with 12 megapixels, providing detailed and clear images of colonies. For image capture, the standard camera app was used in combination with an auto clicker application (e.g. Autoclicker by Simple Design Ltd.). The camera application controls exposure, color, and focus of the camera of the smartphone. Eventually, the auto clicker can be configured to trigger the camera after a specified time to capture an image, while we captured an image every 10 min. For remote access and control of the smartphone further applications (e.g. TeamViewer Host in combination with TemaViewer full client) can be used. Thus, the smartphone can be remotely controlled at any time and from any location, allowing for continuous tracking of images. The images can be stored on the smartphone and eventually transferred to a computer for further analysis.

### Design and construction of SMARTIS

**Figure 1.**
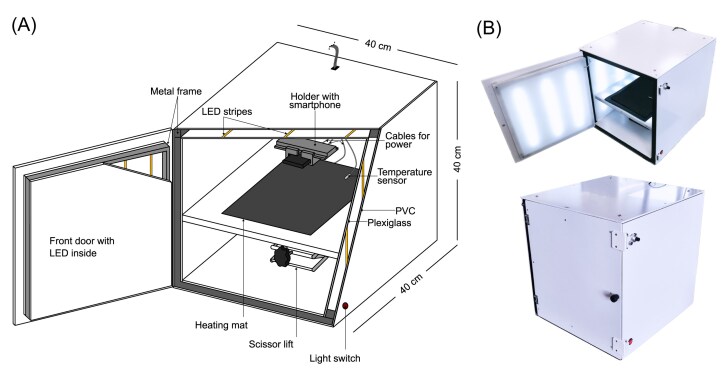
Imaging chamber SMARTIS. (A) Schematic representation of SMARTIS. The walls of SMARTIS consist of plexiglass in which LED lights are installed. Here, a metal frame serves to attach inner and outer panels made out of PVC or Plexiglas. LED lights are turned on using a switch on the outside, while light intensity can be regulated via a tuning control. A smartphone is placed in a holder, while its camera is facing down. The holder can be adjusted to fit any smartphone model. Agar plates can be placed on a heat mat that is manually regulated with a thermostat, while a temperature sensor measures the temperature inside the box. The height of a table that holds the heat mat and the agar plates can be adjusted using a scissor lift. The illustration reflects SMARTIS in a scale of 1:10. (B) Images of the assembled imaging chamber. The top image shows SMARTIS with the front door open, while the bottom image presents a closed view.

SMARTIS is a custom-built imaging chamber designed to facilitate the consistent and reliable capturing of colony growth using a smartphone. The device combines controlled lighting, temperature regulation, and an adjustable platform to optimize the imaging conditions (Fig. 1A and B).

The box is a cubic chamber with dimensions of 40 cm × 40 cm × 40 cm, providing enough space to incubate up to four petri dishes at the same time and consists of multiple panels made of Polyvinyl chloride (PVC) and milky plexiglass, which are attached to a cubic metal frame. Light-emitting diodes (LED) are embedded between the panels and powered by a power supply. Here, the cables are hidden within the gaps of the different panels, while small holes drilled into the plexiglass and PVC route the cables to the power supply, that is attached on the outside of the imaging box. By connecting the power supply to a switch and a tuning control, the LEDs can be turned on and off and dimmed as needed. A heating mat, controlled by a digital thermostat, is integrated into the box to maintain a constant internal temperature of room temperature up to 42°C. A custom-built smartphone holder is installed on the inner ceiling of the imaging box. This holder is adjustable via screws, making it compatible with various smartphone models. A hole in the top panel of the box allows for cable. A table that eventually carries the heating mat and agar plates is mounted on a scissor lift, allowing for height adjustments. The door of the imaging box has an additional metal frame with plexiglass mounted on it, allowing for the integration of additional LED strips behind the panel. The key components required to assemble a SMARTIS, along with their associated costs, are listed in Table 1 and Table S2.

**Table 1.** Components used for SMARTIS with examples and approximate costs.

Component	Example	Approximate cost
Smartphone	Samsung Galaxy S7 mini	170€
Heating mat	Seedling heat mat (Riogoo)	18€
Temperature regulator	Digital wireless temperature controller socket (Ruizhi)	18€
Smartphone cooling	FunCooler 3 Pro	40€
Chamber (this study)	Custom-built (see methods)	379€
	Total:	625€

### Efficiency of transformation assays

*Escherichia coli* BL21-AI cells were transformed with plasmids enabling the production of recombinant Type IV-A1 crRNPs targeting the plasmid pSR14 (see Table S1). Production of crRNPs was induced by the addition of 0.1 mM isopropyl-β-d-thiogalactopyranoside (IPTG) and 0.2% arabinose to the *E. coli* cells, which were then transformed with pSR14, a target vector carrying a matching protospacer and a 5′-AAG-3′ PAM sequence. The pSR15 vector with a nonmatching protospacer served as a negative control. Cells were plated on LB agar plates supplemented with appropriate antibiotics (100 µg/ml ampicillin; 100 µg/ml spectinomycin; 50 µg/ml kanamycin or 34 µg/ml chloramphenicol) and incubated at 37°C. Growth was optionally observed using SMARTIS and transformation efficiency was calculated while normalizing to the negative control. Images of representative LB agar plates were taken using the Gel Stick “Touch” or SMARTIS.

### CRISPR interference assays

*Escherichia coli* BL21-AI cells able to produce recombinant Type IV-A1 crRNPs using one or two plasmids and targeting the *lacZ* gene (see Table S1) were cultured for 5 h at 37°C in 3 ml of LB supplemented with respective antibiotics (100 µg/ml ampicillin; 100 µg/ml spectinomycin or 50 µg/ml kanamycin). Cultures were diluted to an OD_600nm_ of 2 and a dilution series up to 10^–5^ was prepared. Cells were transferred onto LB agar plates supplemented with appropriate antibiotics, 40 µg/ml X-gal, 0.2% arabinose, and 1 mM IPTG. Plates were incubated at 37°C. Images were analyzed using OpenCFU 3.8-BETA (Geissmann 2013), while the color filter was set to a hue angle of 0–80 to distinguish white and blue colonies. Images of representative LB agar plates were taken using a smartphone (Samsung Galaxy S7 mini), while a microscope for smartphone was used to enhance single colonies.

### Analysis of time-lapse images

The automated image analysis tool ColTapp was used to analyze time-lapse images of agar plates (Bär et al. 2020). For further analysis R Studio (https://posit.co/) was used, while graphs were generated using GraphPad Prism (v8.0.2).

## Results and discussion

### Observation of heterogenous colonies with Type IV-A1 crRNP production

**Figure 2.**
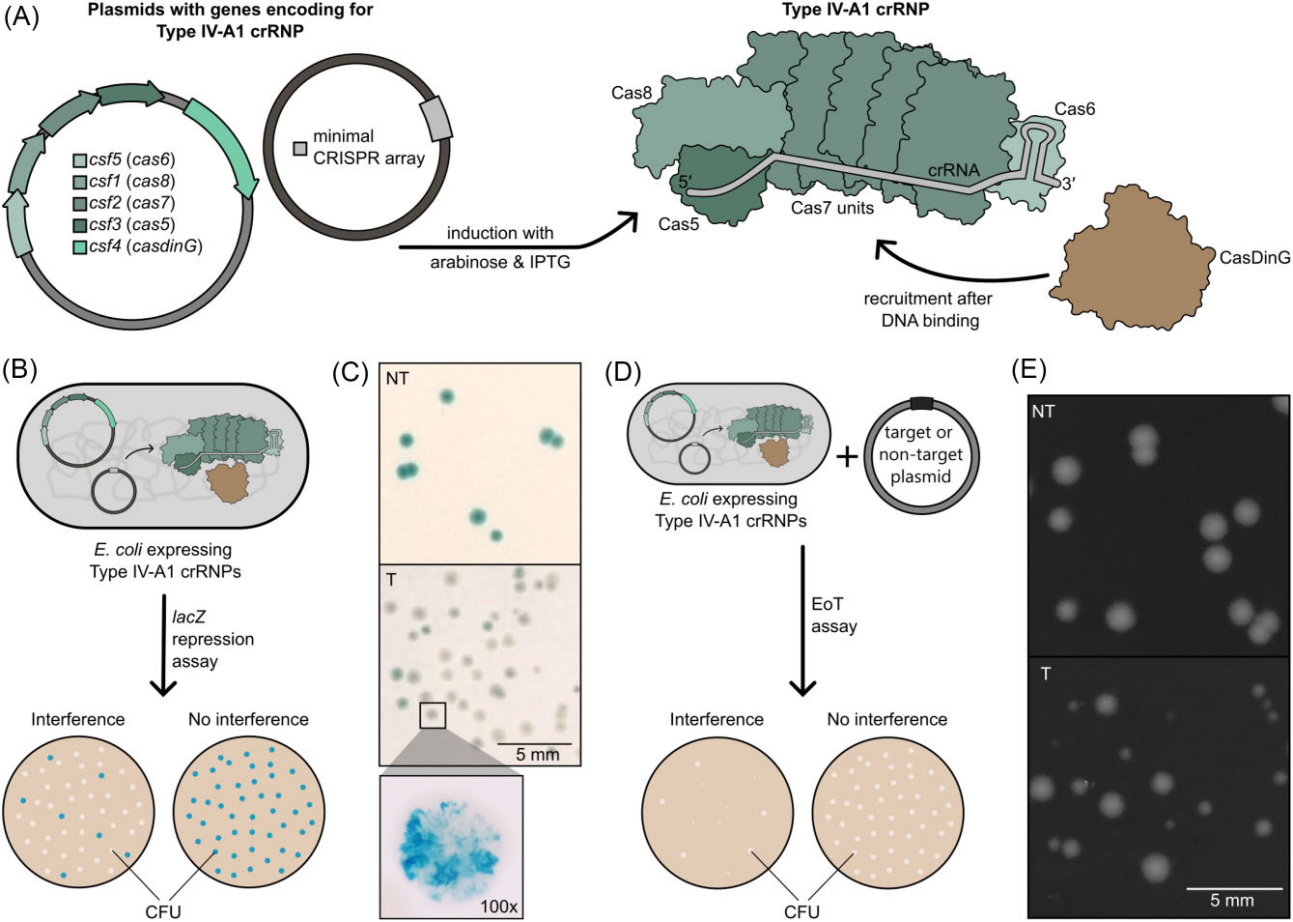
Type IV-A1 CRISPRi assays result in heterogenous colonies. (A) Plasmids contain genes encoding the Type IV-A1 crRNP proteins (Cas5, Cas6, Cas8, Cas7, and CasDinG) and a minimal CRISPR array (repeat-spacer-repeat). After induction with arabinose and IPTG proteins associate to Type IV-A1 crRNPs with synthetic crRNAs, while recruitment of CasDinG occurs after DNA binding. (B) Schematic of a *lacZ*-targeting assay. *Escherichia coli* cells express Type IV-A1 crRNPs targeting the genomic gene *lacZ*. Colonies can be visually differentiated by color (blue or white). Blue color indicates intact *lacZ* and refers to ineffective CRISPR-Cas activity (no interference), while white colonies indicate inhibition of *lacZ* and, thus, effective targeting (interference). (C) Type IV-A1 crRNPs with a targeting crRNA against *lacZ* (T) or a nontargeting crRNA with a random spacer (NT) are expressed, while representative images of LB agar plates of a 10^–4^ dilution and a single colony after a *lacZ* targeting assay are shown. The enhanced image of an individual colony shows uneven distribution of blue and white color within a single colony. (D) Schematic of an efficiency of transformation assay, while *E. coli* cells expressing Type IV-A1 crRNPs are transformed with either a target (T) or nontarget plasmid (NT). CFU are counted and calculated efficiency of transformation indicates the efficiency of Type IV-A1 crRNPs. Efficient targeting results in a reduced amount of CFU/ml (interference), while inefficient or no targeting results in a high number of CFUs (no interference). (E) Representative LB agar plates with CFUs after an EoT assay, while a 10^–4^ the nontargeting and a 10^–1^ dilution of the targeting control are shown.

Previous studies indicated efficient plasmid interference and self-targeting activity when using a recombinant Type IV-A1 CRISPR-Cas system in *E. coli*, while crRNPs were expressed using a two-plasmid approach. Here, essential *cas* genes and a minimal CRISPR array are located on two different plasmids (Crowley et al. 2019, Guo et al. 2022). In this study, we examined plasmid- and genome-targeting activity in more detail to analyze colony heterogeneity during Type IV-A1 interference. We first reproduced the two-plasmid approach for the expression of recombinant Type IV-A1 crRNPs in *E. coli* BL21-AI. Thus, we used a pETDuet-1™ derivative encoding for all Type IV-A1 *cas* genes from *P. oleovorans* DSM 1045 and a pCDFDuet-1™ derivative encoding for a synthetic crRNA (see Table S1). After introduction of the plasmids in *E. coli* cells, Type IV-A1 crRNP formation was induced with arabinose and IPTG (Fig. 2A).

We first performed *lacZ* targeting assays, while a synthetic crRNA is targeting the genomic gene *lacZ* in *E. coli* BL21-AI as previously described (Guo et al. 2022). Successful downregulation of *lacZ* expression by Type IV-A1 CRISPR-Cas activity eventually leads to the appearance of white colonies on LB plates supplemented with X-Gal (Fig. 2B), while a nontargeting (NT) or dysfunctional system results in only blue colonies. Using the described expression system, we obtained blue colonies when using a NT system, and blue and white colonies after targeting the *lacZ* gene (Fig. 2C). We were also able to produce individual colonies showing a mixture of blue- and white-colored sections. Thus, we further examined the heterogeneous colonies and observed a cloud-like, irregular pattern with varying shades of blue (Fig. 2C). While white colonies typically indicate effective targeting and repression of *lacZ*, the occurrence of mixed blue–white colonies suggests incomplete or uneven targeting efficiency across the bacterial population or unequal inheritance of the expression plasmids. This variability could arise from differences in crRNP assembly, plasmid replication, or segregation during cell division, leading to subpopulations within individual colonies with varying plasmid numbers or targeting activity (Tomoiaga et al. 2022). However, heterogeneity of colonies was primarily associated with *lacZ* genome targeting in the previous study (Guo et al. 2022). Therefore, we further analyzed the activity of crRNPs targeting a plasmid, aiming to uncover whether plasmid targeting results in similar variability of colony growth.

We performed EoT assays using either a target (T) or a nontarget (NT) plasmid, that carry a matching protospacer with a PAM (5′-AAG-3′) or a random spacer sequence, respectively (Fig. 2D). The introduction of a target plasmid led to a significant decreased efficiency of transformation (EoT) in comparison to the use of a nontarget plasmid (Fig. S2), comparable to previous studies (Crowley et al. 2019, Guo et al. 2022). Notably, we observed heterogeneity of colony sizes when crRNPs effectively targeted the plasmid, while colony sizes appeared homogenous when a nontarget plasmid was introduced (Fig. 2E). However, we were not able to determine at what stage heterogeneous colonies emerged using only end-point images. Thus, we monitored the bacterial growth over time using SMARTIS, which allows for a more detailed analysis of colony growth dynamics.

### Heterogeneity of colonies occurs early in growth

**Figure 3.**
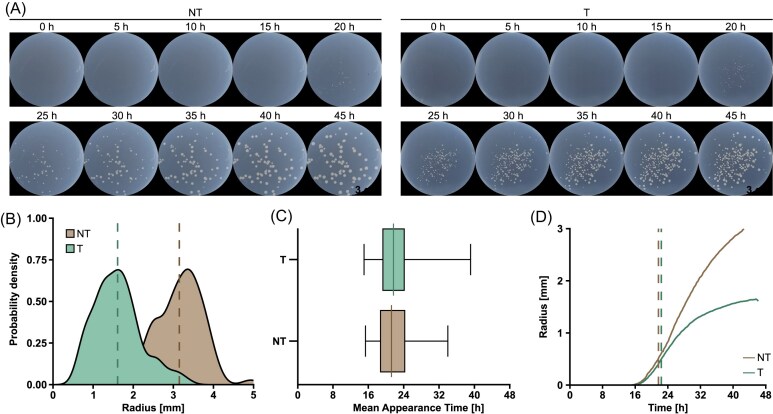
SMARTIS reveals growth dynamics of cells after an EoT assay. (A) Colony growth on respective plates of different controls after an EoT assay that was performed according to Fig. 1(D). Plates of a 10^–3^ dilution of a NT control and a 10^–1^ dilution of a targeting control (T) grew at 37°C and were observed for 46 h using SMARTIS. Different dilutions were used to enable further analysis, using plates with 25–300 colonies. Images were captured every 10 min. (B–D) Analyses of end-point and time-lapse images of the respective plates shown in (A) using ColTapp (Bär et al. 2020). (B) Distribution of colony sizes (radius) after 46 h of growth. Mean radius of controls are indicated as dashed lines. Mean radius significantly differs among the different controls (unpaired *t*-test, *P*-value < .0001). (C) Box plot of appearance times of colonies of the different controls (T and NT). The center line of the box plot represents the mean appearance time, with the left and right borders of the box marking the minimum and maximum, respectively. The number of colonies used for calculations differs among the different controls with *n* = 76 for NT and *n* = 222 for T, while appearance time does not significantly differ (unpaired *t*-test, *P*-value = .0880). (D) Mean colony growth over time of the different controls. Dashed lines indicate mean appearance times.

To ensure stable colony growth at a specific temperature, we first examined the temperature within the custom-built box over time. SMARTIS was set to 37°C, and temperature measurements were recorded over a 24-h period. No significant temperature fluctuations were detected during our observation, with an average temperature of 36.96°C and a preheating time of ∼20 min (Fig. S2). Thus, the system would enable bacterial growth within a closed environment at a constant temperature. We, therefore tracked colony growth after performing an EoT assay with the two-plasmid approach in *E. coli* BL21-AI cells. Up to four plates of different dilutions were simultaneously incubated at 37°C and observed using SMARTIS, while the smartphone was set to capture an image every 10 min within 46 h. SMARTIS allowed for high-resolution images of the plates, ensuring clear visualization of colony growth and morphology. We did not encounter any technical issues throughout the time-lapse imaging process, allowing for consistent data collection. Over the 46-h period, we successfully captured 277 images, providing a time-lapse sequence of colony development (Fig. 3A, Video 1). The images already revealed that colonies in the T and the NT control appeared at similar time points, indicating that the initiation of colony formation was not significantly influenced by experimental conditions. However, colonies in T resulted in heterogeneity of colony sizes, suggesting that individual colonies grew with varying growth rates, likely because of the expressed crRNPs targeting the plasmid. To quantify colony sizes and growth dynamics, we used the recently developed software ColTapp (Bär et al. 2020) for time-lapse image analysis, ensuring accurate data processing and facilitating the identification of growth patterns that are not immediately apparent through visual inspections of the images. We first determined the size of individual colonies using the end-point images after 46 h of growth. Here, we observed a clear distribution of colony sizes among the different controls, while colonies of the NT control generally appeared larger compared to the targeting control (Fig. 3B). Notably, the maximum colony size observed in the targeting control was very similar to the average colony size in the NT control, indicating generally better growth when there is no targeting. This finding already suggests significant differences in growth dynamics between the two conditions, underscoring the influence of targeting activity or plasmid properties on bacterial colony development (Table S3). We analyzed the image sequences to examine the appearance times of individual colonies and to evaluate the growth across the different controls. Here, the appearance time refers to the first time point a colony is macroscopically visible and can be detected by the used analysis software ColTapp (Bär et al. 2020).

Mean appearance times of colonies only showed minor differences among the controls, while the first colonies appeared after ~15 h (Fig. 3C and D, Table S3). Here, we did not observe significant variations between the different targeting controls. However, the last occurring colonies emerged at slightly different times: When nontargeting, the final colony appeared after 34 h, whereas the last colony of the targeting control started showing after 39 h of growth (Sanchez-Londono et al. 2024). Colonies of the targeting control exhibited an average colony extension of ~35 µm/h, while colonies with a nontarget plasmid grew at a rate of about 68 µm/h, which is nearly double the colony extension of the targeting control (Table S3). We assumed that the differing colony extensions are influenced by crRNP activity or varying numbers of target or nontarget plasmids. However, to exclude a correlation of the observed heterogeneity with the used expression system, we designed and examined different plasmids expressing Type IV-A1 crRNPs.

### Impact of different crRNP expression plasmids on colony heterogeneity

The *cas* genes were cloned with a CRISPR array containing a synthetic spacer designed with target sites for the type IIS restriction enzyme BseRI, following a recent protocol (Rust and Randau 2024). The approach enhances the adaptability of spacer sequences, allowing for the flexible design of synthetic spacers. Thus, plasmid derivatives of different plasmids were constructed to carry spacer sequences for either EoT or *lacZ* targeting assays (Fig. S3). The used plasmids differ in copy numbers and resistance markers, while we used plasmids of the Duet™ expression system (Novagen), suitable for expression of potentially toxic protein complexes (see Table S1). The theoretical copy numbers of the plasmids pCDFDuet-1, pETDuet-1, and pRSFDuet-1 are 20, 40, and 100, respectively (Yang et al. 2019). Notably, transformation of empty plasmid backbones did not generate heterogeneity of colonies, suggesting that plasmid features alone do not induce variability in colony growth and are not the cause for heterogenous colony sizes or colors.

**Figure 4.**
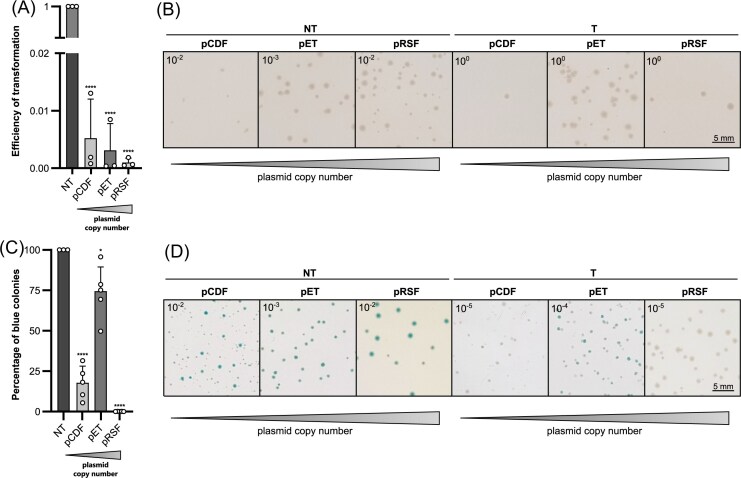
Influence of different expression systems on colony heterogeneity. (A) Results of EoT assays using different expression plasmids (pCDF, pET, or pRSF). A target plasmid with a matching protospacer was used as a targeting control (T) in combination of each plasmid, while a nontarget plasmid, carrying a random protospacers, refers to the NT control. EoT is calculated using the ratio of targeting and NT control of each plasmid. (B) End-point images of representative plates with colonies after EoT assays with pET, pCDF, or pRSF. Plates of different dilutions ranging from 10^0^ to 10^–3^ are shown and indicated in the figure, while plates with 25–300 CFUs were used for calculations in A. (C) Results of *lacZ*-targeting assays using pCDF, pET, or pRSF. Blue–white screening of the cells resulted in different percentages of blue colonies depending on the used expression plasmid, while the NT control showed only blue colonies in case of all plasmids used. (D) End-point images of representative colonies of blue–white screening after *lacZ*-targeting assays with pET, pCDF, or pRSF. Plasmids expressing either a targeting crRNP (T) or a NT crRNP were used. Heterogeneity of colony sizes is visible for pCDF, while homogenous colony sizes appeared in case of pET or pRSF. Plates of different dilutions ranging from 10^–2^ to 10^–5^ are shown and indicated in the figure, while plates with 25–300 CFUs were used for calculations in C. EoT and *lacZ*-targeting assays were performed as described in Fig. 1. Data are presented as mean values +SD. *P*-values were calculated using an unpaired *t*-test. (A and C: ^****^  *P* < .0001; C: * *P* = .0379). Video 1. Growth of different controls after an EoT assay. Time-lapse video of colony growth on respective plates of different controls after an EoT assay, performed according to Fig. 1(D). Plates of a 10^−3^ dilution of the NT control and a 10^−1^ dilution of the targeting control (T) were incubated at 37°C and monitored for 46 h using SMARTIS. The camera of the smartphone was set to capture an image every 10 min, resulting in a total of 277 images. Images were used to generate a video sequence, while each image is shown for 0.05 s.

Following the transformation of *E. coli* BL21-AI cells with the adapted plasmids and performing EoT and *lacZ* targeting assays, end-point images of the resulting plates were analyzed. All plasmid derivatives resulted in efficient plasmid targeting as shown by a reduced EoT (Fig. 4A). However, we observed differences in colony sizes between the plasmid variants, which we also noticed in the negative controls of pCDF or pRSF (Fig. 4B). This observation contradicts our initial hypothesis that heterogeneity in colony sizes is primarily driven by the activity of targeting crRNPs, which we assumed would cause variability in colony extensions due to plasmid loss and, thus, uneven inheritance of plasmids. We further examined the different plasmids in genome targeting assays. Targeting of *lacZ* yielded different numbers of white and blue colonies depending on the plasmid used. While pRSF led to the highest efficiency with almost 100% white colonies among the assays, pET resulted in a significant decreased efficiency (Fig. 4C). The use of pCDF resulted, besides blue and white colonies, in highly heterogenous colony sizes, which were also visible in the NT control. The heterogeneous growth of colonies expressing Type IV-A1 crRNPs with pCDF may lead to misinterpretation of results. While we used OpenCFU (Geissmann 2013) for colony counting and analysis following *lacZ* targeting assays the software was unable to accurately count very small colonies. Therefore, the results of this specific construct should be treated with caution. However, strong variations in colony sizes were not observed when using pET or pRSF, while targeting using the pET plasmid again resulted in mixed blue–white colonies (Fig. 4D). Notably, numbers of CFUs were decreased when using the plasmid pCDF for *lacZ* targeting, which was not the case when using it for plasmid-targeting. However, the results of the EoT assay with pCDF show no significant difference in CFU numbers between nontargeting and targeting, possibly due to heterogeneous growth (Fig. 4B, Fig. S4). Thus, the intermediate copy number plasmid pET and high copy number plasmid pRSF appear to be more suitable for expressing crRNPs in the context of genome targeting, with the copy number of the respective plasmid likely being a key factor influencing their efficiencies. Here, pET may provide moderate levels of crRNP expression while minimizing metabolic burden and, thus, causing an increasing number of colonies able to evade CRISPR-Cas activity. In contrast, pRSF typically exceeds 100 copies per cell, which can drive higher crRNP expression but may impose greater metabolic stress on the host cells. We also observed a potential impact on cell viability when examining the total number of CFU for the different constructs. Specifically, numbers of CFUs of cells using the pRSF expression plasmid were slightly lower compared to those expressing crRNPs on the pET plasmid. This reduction may reflect the increased metabolic burden associated with the higher copy number of pRSF, which could affect overall cell growth and viability. The differences suggest a potential correlation between the copy number of an expression plasmid and genome targeting efficiency, where higher plasmid copy numbers may enhance targeting efficiency but also increase cellular resource demands.

Overall, our findings underscore the importance of careful consideration when selecting expression systems for CRISPRi-based applications, as differences in growth dynamics may influence the interpretation of results.

## Conclusion

In our study, we demonstrate how SMARTIS enables high-resolution, time-lapse imaging of bacterial colonies, providing a powerful and cost-effective tool to identify and track heterogeneous growth phenotypes. By applying this method, we observed that variability in colony expansion is influenced by multiple factors, including plasmid expression systems, antibiotic selection, and Type IV-A1 CRISPR-mediated interference. Here, the formation of crRNPs and their subsequent scanning activity for a complementary target DNA may impose a significant metabolic burden on bacterial cells, influencing their viability. The continuous engagement with DNA during this search process, even in the absence of a target, could interfere with essential cellular processes, such as replication or transcription, thereby reducing cell fitness or viability. In the context of plasmid targeting, this activity can lead to undesirable inhibition of plasmid replication, resulting in the uneven distribution of plasmids among daughter cells. Consequently, subpopulations within a colony may carry varying numbers of plasmids, leading to differences in cellular fitness and contributing to heterogeneity (Hülter et al. 2020).

We used different expression plasmids that varied in antibiotic resistance and origins of replication, leading to differences in plasmid copy number and replication mechanisms. We cannot exclude that these variations may further influence the efficiency of crRNP-mediated interference, while the selective pressure of different antibiotics may significantly affect bacterial growth. Even in strains that are resistant, the metabolic burden associated with maintaining resistance mechanisms can inhibit growth (Chowdhury and Findlay 2023). This effect can be intensified when multiple antibiotics are used simultaneously, as seen in experiments involving EoT assays, where cells are exposed to two or three antibiotics for selection. The cumulative stress from maintaining resistance to different drugs and expression of Type IV-A1 crRNPs can lead to uneven resource allocation within the bacterial population, creating variations in colony extensions and fitness among individual cells. The unique mechanism of Type IV-A1 crRNPs may further contribute to heterogeneous colony growth. In contrast to dCas9, which exerts a localized interference effect, the Type IV-A1 system can downregulate an extended region up- or downstream of the target site (Qi et al. 2013, Sanchez-Londono et al. 2024). Consequently, when targeting genomic DNA, Type IV-A1 crRNPs can influence adjacent genes, which may explain the observed differences in interference efficiencies between plasmid- and genome targeting for different expression systems.

Overall, our study emphasizes the importance of understanding how plasmid properties and antibiotic selection pressures contribute to heterogeneity in bacterial colonies, while highlighting the potential of SMARTIS as a powerful tool to unravel the complexities of bacterial growth. By establishing SMARTIS, we provide a user-friendly, cost-effective method for high-resolution, time-lapse imaging of bacterial colonies, suitable for a variety of research questions that benefit from automated assessment of colony phenotypes over time.

## Supplementary Material

uqaf006_Supplemental_Files

## Data Availability

The data supporting this article can be found within the article itself and its supplementary material.
