# Supplementary material for: Real-time imaging of bacterial colony growth dynamics for cells with Type IV-A1 CRISPR-Cas activity
Source: Microlife. 2025 Apr 1;6:uqaf006. doi: 10.1093/femsml/uqaf006 (PMC11995694; doi:10.1093/femsml/uqaf006)
Supplement: uqaf006_Supplemental_Files [file uqaf006_supplemental_files.zip › Supplementary SMARTIS submit revision 2.pdf]

Supplementary Material

Real-time imaging of bacterial colony growth dynamics for cells with Type IV-A1 CRISPR-Cas activity

Selina Rust [1], Lennart Randau [1,2*]

[1] Prokaryotic RNA Biology, Department of Biology, Philipps-Universität Marburg, Marburg, Germany

[2] SYNMIKRO, Center for Synthetic Microbiology, Marburg, Germany

Supplementary material content

Supplementary Tables:

Supplementary Table S1. Plasmids used in this study.

Supplementary Table S2. Specific materials used for the construction of the chamber of the SMARTIS.

Supplementary Table S3. Results of image sequence analysis.

Supplementary Figures:

Supplementary Figure S1. Results of EoT assays using a two-plasmid Type IV crRNP expression system.

Supplementary Figure S2. Temperature measurement over time using SMARTIS.

Supplementary Figure S3. Schematic representation of different expression plasmids.

Supplementary Figure S4. Total numbers of colony forming units (CFU) after EoT and *lacZ* targeting assays.

Supplementary Table S1. Plasmids used in this study.

Plasmid	Derivative of	Insertion	Reference
pSR13	pETDuet-1™	*csf5*, *csf1*, *csf2*, *csf3* and *csf4* of the Type IV-A1 CRISPR-Cas system of *P. oleovorans* DSM1045	(Guo et al., 2022)
pSR14	pACYCDuet-1™	target plasmid; protospacer sequence matching with pSR24	(Guo et al., 2022)
pSR15	pACYCDuet-1™	non-target plasmid; random protospacer sequence	(Guo et al., 2022)
pSR24	pCDFDuet-1™	minimal CRISPR array; repeats of Type IV-A1 CRISPR-Cas system of *P. oleovorans* DSM1045; synthetic spacer sequence (5′-CATCCAAGTTACGCATCAGATTCGAGACGCGA-3′) targeting pSR14	(Guo et al., 2022)
pSR54	pCDFDuet-1™	minimal CRISPR array; repeats of Type IV-A1 CRISPR-Cas system of *P. oleovorans* DSM1045; synthetic spacer sequence targeting *lacZ* (5′-ATCGCACTCCAGCCAGCTTTCCGGCACCGCTT-3′)	(Guo et al., 2022)
pSR56	pCDFDuet-1™	minimal CRISPR array; repeats of Type IV-A1 CRISPR-Cas system of *P. oleovorans* DSM1045; synthetic non-targeting spacer sequence; **BseRI recognition sites** (5′-ATGTGACT**CTCCTC**CGAA**GAGGAG**GTAAGTAC-3′)	(Guo et al., 2022)
pSR64	pCDFDuet-1™	*csf5*, *csf1*, *csf2*, *csf3* and *csf4* of the Type IV-A1 CRISPR-Cas system of *P. oleovorans* DSM1045 and minimal CRISPR array with synthetic non-targeting spacer sequence; **BseRI recognition sites** (5′-ATGTGACT**CTCCTC**CGAA**GAGGAG**GTAAGTAC-3′)	This study
pSR65	pSR64	refers to pCDF; synthetic spacer sequence targeting *lacZ*; (5′-ATCGCACTCCAGCCAGCTTTCCGGCACCGCTT-3′)	This study
pSR76	pETDuet-1™	*csf5*, *csf1*, *csf2*, *csf3* and *csf4* of the Type IV-A1 CRISPR-Cas system of *P. oleovorans* DSM1045 and minimal CRISPR array with synthetic non-targeting spacer sequence; **BseRI recognition sites** (5′- ATGTGACT**CTCCTC**CGAA**GAGGAG**GTAAGTAC-3′)	This study
pSR77	pRSFDuet-1™	*csf5*, *csf1*, *csf2*, *csf3* and *csf4* of the Type IV-A1 CRISPR-Cas system of *P. oleovorans* DSM1045 and minimal CRISPR array with synthetic non-targeting spacer sequence; **BseRI recognition sites** (5′-ATGTGACT**CTCCTC**CGAA**GAGGAG**GTAAGTAC-3′)	This study
pSR79	pSR76	refers to pET; synthetic spacer sequence targeting pSR14; (5′- CATCCAAGTTACGCATCAGATTCGAGACGCGA-3′)	This study
pSR80	pSR77	refers to pRSF; synthetic spacer sequence targeting pSR14; (5′- CATCCAAGTTACGCATCAGATTCGAGACGCGA-3′)	This study
pSR81	pSR64	refers to pCDF; synthetic spacer sequence targeting pSR14; (5′- CATCCAAGTTACGCATCAGATTCGAGACGCGA-3′)	This study
pSR101	pSR76	refers to pET; synthetic spacer sequence targeting *lacZ*; (5′-ATCGCACTCCAGCCAGCTTTCCGGCACCGCTT-3′)	This study
pSR102	pSR77	refers to pRSF; synthetic spacer sequence targeting *lacZ*; (5′-ATCGCACTCCAGCCAGCTTTCCGGCACCGCTT-3′)	This study

Supplementary Table S2. Specific materials used for the construction of the chamber of the SMARTIS.

Materials	Amount used	Example cost
LEDs with power supply	1 piece	62.00 € /piece
Polyvinyl chloride (PVC) black (30 mm)	1 piece	8.00 €/ piece
Aluminum tube (20 x 20 x 2 mm)	6 m	5.50 €/ meter
PVC white (4 mm)	5 kg	20.00 €/ kilogram
Acrylic glass white milky (500 x 500 mm)	5 pieces	23.00 €/ piece
Silicone white	1 piece	5.70 €/ piece
Hinges (40 x 60 mm)	2 pieces	2.50 €/ piece
Scissor lift	1 piece	38.00 €/ piece
Different screws	38 pieces	0.31 €/ piece
	**Total**	**378.50 €**

Supplementary Table S3. Results of image sequence analysis. ColTapp (Bär et al., 2020) and RStudio (posit team) were used to determine colony sizes (radius) at the end, appearance times and colony extensions of different controls.

Control	Average endpoint radius [mm]	Minimum endpoint radius [mm]	Maximum endpoint radius [mm]	Average appearance time [hours]	Minimum appearance time [hours]	Maximum appearance time [hours]	Average extension [µm/hour]
C-	3.15	1.64	4.93	21.74	15.30	39.17	68.45
C+	1.61	0.59	3.40	22.40	15.00	34.00	35.02

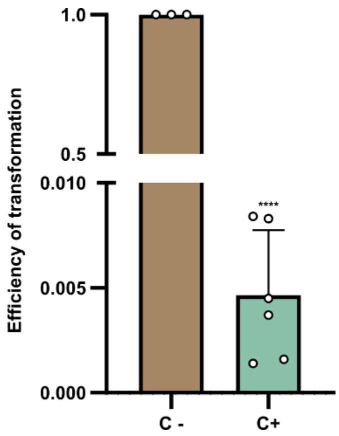

Supplementary Figure S1. Results of EoT assays using a two-plasmid Type IV crRNP expression system. The expression plasmids contain genes encoding the Type IV-A1 crRNP proteins (pETDuet-1™ derivative) and a minimal CRISPR array (pCDFDuet-1™ derivative). After induction with arabinose and IPTG, a target plasmid (C+) with a matching protospacer and adjacent motif (5′-AAG-3′) or a non-target plasmid (C-) with a random protospacer is introduced. Efficient targeting refers to a downregulation of efficiency of transformation. Data are presented as mean values +SD. P-values were calculated using an unpaired t-test. (**** P < 0.0001).

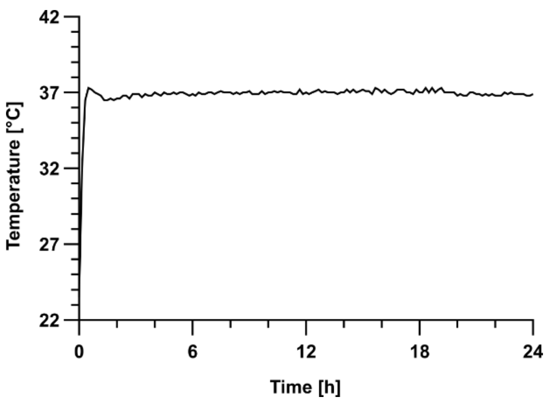

Supplementary Figure S2. Temperature measurement over time using SMARTIS. Temperature was measured every 10 minutes within a period of 24 hours. Average temperature of 36.96 °C was calculated with all data points after 0.33 hours.

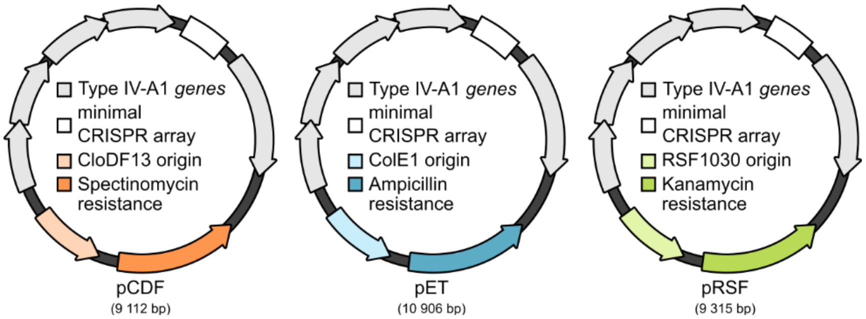

Supplementary Figure S3. Schematic representation of different expression plasmids. All plasmids carry genes encoding proteins for assembly of Type IV-A1 crRNPs, while a minimal CRISPR array contains a single spacer sequence flanked by identical repeats. Plasmids vary in sizes, replication origin and encoded resistance cassette.

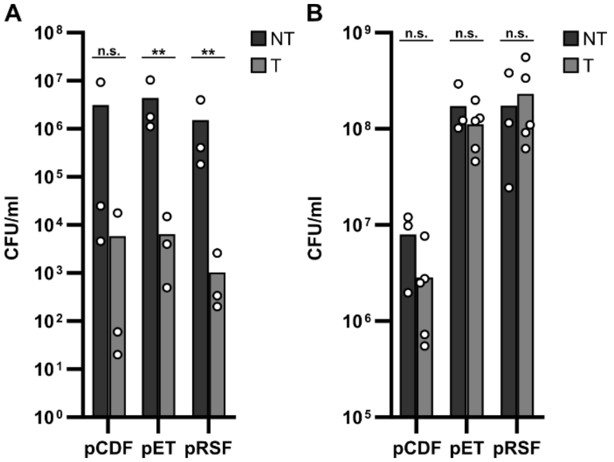

Supplementary Figure S4. Total numbers of colony forming units (CFU) after EoT and *lacZ* targeting assays. CFU/ml were calculated using dilutions that resulted in 25 to 300 CFU (dilutions 10^{-3} 10^{-5}, depending on the construct). (**A**) CFU/ml after EoT assays. A target plasmid with a matching protospacer refers to the targeting control (T), while a non-target plasmid, carrying a random protospacers, refers to the non-targeting control (NT). (**B**) CFU/ml after lacZ targeting assays. The targeting control (T) refers to the expression plasmid able to target the gene lacZ, while the non-targeting control (NT) carries a random spacer sequence. P-values were calculated using an unpaired t-test (** $P < 0.01$, n.s. = non significant).

Supplementary References

Bär, J., Boumasmoud, M., Kouyos, R. D., Zinkernagel, A. S., & Vulin, C. (2020). Efficient microbial colony growth dynamics quantification with ColTapp, an automated image analysis application. *Scientific Reports*, *10*(1), 16084. https://doi.org/10.1038/s41598-020-72979-4

Guo, X., Sanchez-Londono, M., Gomes-Filho, J. V., Hernandez-Tamayo, R., Rust, S., Immelmann, L. M., Schäfer, P., Wiegel, J., Graumann, P. L., & Randau, L. (2022). Characterization of the self-targeting Type IV CRISPR interference system in *Pseudomonas oleovorans*. *Nature Microbiology*, *7*(11), 1870–1878. https://doi.org/10.1038/s41564-022-01229-2
